# Learning by Social Interactions: Insights into Observational Learning in Autism Spectrum Disorder

**DOI:** 10.3390/brainsci16040357

**Published:** 2026-03-26

**Authors:** Tiziana Iaquinta, Luca Pullano, Elena Commodari, Francesca Foti

**Affiliations:** 1 Department of Medical and Surgical Sciences, “Magna Graecia” University of Catanzaro, 88100 Catanzaro, Italy; luca.pullano@unicz.it; 2 Department of Educational Sciences, University of Catania, 95124 Catania, Italy; elena.commodari@unict.it

**Keywords:** learning by observation, social learning, social environments, social and non-social observational learning, video modeling, generalization, imitation, small-group instruction, autism spectrum disorder, neurodevelopmental disorders

## Abstract

**Highlights:**

**What are the main findings?**

- Individuals with ASD may be able to learn by observing others, especially if they are taught the prerequisites for observational learning to occur.
- Observation may provide an effective way to expand the typically restricted and circumscribed interests of children with ASD, as well as to increase emotion recognition skills.

**What are the implications of the main findings?**

- These findings have significant clinical, educational, and social implications supporting the use of observational learning procedures to improve some of the core deficits of ASD and to reduce reliance on expensive one-on-one tutoring.
- These findings offer new insights into the interplay between observational learning and social information processing and how the atypical ASD social functioning modulates social and non-social observational learning.

**Abstract:**

**Background/Objectives**: Observational learning allows people to acquire new skills by observing the actions of others embedded in their social environment. From childhood, observational learning is a central process in human cognitive development, playing a crucial role in the acquisition of complex skills. Children and adults with autism spectrum disorder (ASD) often exhibit deficits in what are considered prerequisites for observational learning to occur (i.e., attending, imitation, delayed imitation, consequence discrimination). Considering this, the present review examined the literature on the complex and timely question of whether individuals with ASD can learn by observation, while accounting for the social versus non-social nature/content of the tasks. **Methods**: This work was a narrative review aimed at providing an overview of published studies in which observational learning was analyzed in individuals with ASD. Twenty-two studies met the inclusion criteria and were eligible for this review. **Results**: The core findings indicate that individuals with ASD may be able to learn by observing others, especially when taught the prerequisites for observational learning. Furthermore, the findings indicate that observation may be an effective way to expand the typically restricted and circumscribed interests of children with ASD and to increase emotion recognition skills. **Conclusions**: Overall, these findings have significant educational, clinical, social, and economic implications, supporting the use of observational learning strategies for both social and non-social skills to reduce reliance on expensive one-on-one teaching and to address some of the core deficits of ASD.

## 1. Introduction

Humans are intrinsically social agents [1]. Social interactions with other people in daily life are powerful means of learning how to interact with the world [2]. What humans learn through their social lives is called social learning. An important form of social learning is represented by observational learning [3,4], which refers to the process of acquiring new skills by observing other people’s behaviors and the contingencies of those behaviors. Importantly, through observational learning, humans can reduce the time and the attempts to learn complex actions and behaviors [2,5]. From childhood, learning new skills by observing adults or peers is a central process in human cognitive development [6]; indeed, observational learning plays a crucial role in the acquisition of complex abilities such as language, social responsiveness, and the use of instruments to get things done, thus representing a powerful social learning mechanism [6,7,8]. Observation is also involved in the acquisition of academic skills [9] and can be an effective instructional approach in reading, writing, and other academic competencies [10]. Interestingly, recent studies have demonstrated that observation also promotes earlier acquisition of high-level spatial skills in typically developed (TD) children [11,12] and is a powerful way to facilitate the acquisition of complex behaviors also in neurodevelopmental disorders such as Williams or Prader–Willi syndromes [13,14,15].

Developmental research indicates that the capacity to learn from observation is a gradual and complex process in typical development [16,17]. Indeed, to learn by observation, infants need to attend to and observe the model, understand others’ actions and anticipate the effect of the observed action, and, after a delay, match some properties of the observed behavior. Thus, attending, imitating, and understanding contingencies are specific prerequisite skills for learning by observation.

Learning by observing others has significant educational, economic, and social implications. Indeed, if one can learn by observing the consequences delivered to others, this can reduce instructional time and financial costs associated with intensive instruction, and lead to the acquisition of socially relevant behavior, thereby increasing opportunities for social integration [18]. In this regard, it is important to note that an essential skill for successful inclusion in social and educational contexts is the ability to learn by observing others.

The specific features of observational learning, together with its significant implications, raise crucial questions. Can this type of learning be expected in the presence of autism spectrum disorder (ASD)? Can observational learning in individuals with ASD be modulated by the social versus non-social nature and content of the task to be learned?

Children and adults with ASD have difficulty participating in social environments and show considerable difficulties in communication, social skills, and repetitive or stereotypic behaviors based on the *Diagnostic and Statistical Manual of Mental Disorders* [19]. More specifically, research has demonstrated that children and adults with ASD display significant deficits in these fundamental skills necessary for observational learning [20] and in encoding relevant information from demonstrations [21]. Indeed, deficits in imitation, consequence discrimination, and attention, such as poor or inconsistent eye contact, inability to follow eye gaze, failure to orient to toys or materials, and failure to engage in joint attention [22,23,24,25,26], are among the core diagnostic indicators of autism. In addition, the monitoring of social activities is disrupted early in the developmental progression of autism, limiting subsequent abilities for observational learning [27]. The crucial role of observational learning in human development, combined with the deficits observed in individuals with ASD described above, provides the theoretical and applied background for the scientific literature addressing observational learning in ASD.

Considering this, the present review examined the literature on the complex and timely question of whether individuals with ASD can learn by observation, providing an overview of existing studies that examine this crucial topic with significant educational, clinical, social, and economic implications. Moreover, given that ASD is diagnosed as predominantly involving social interaction difficulties—implying reduced social capacities—the existing literature will be examined by considering the social versus non-social nature and content of the tasks. Accordingly, this review will contribute to a better understanding of the mechanisms underlying observational learning and clarify a possible dissociation between social and non-social observational learning, offering new insights into the interplay between observational learning and social information processing and how the atypical ASD social functioning may modulate observational learning. Crucially, these findings could lead to increased social and educational opportunities, thereby reducing reliance on costly one-to-one tutoring.

## 2. Materials and Methods

### 2.1. Literature Search and Study Selection

This work was a narrative review aimed at providing an overview of published studies in which observational learning was analyzed in individuals with ASD. A literature search was conducted according to the following criteria: (a) filter time: from January 2000 through April 2025; (b) database: PubMed; (c) language: English; (d) keywords: terms used were (“autism” OR “autism spectrum disorder” OR “autism spectrum condition”) AND (“observational learning” OR “learning by observation”); (e) key terms were searched in the title and abstract; (f) population: individuals with ASD without age restrictions; (g) the papers’ selection was based on critical reading. The exclusion criteria were: (a) animal model; (b) reviews, conference abstracts, book chapters, or editorials.

The titles and abstracts of all retrieved articles were reviewed for eligibility, applying the inclusion and exclusion criteria mentioned above. When the information provided in the title and abstract was insufficient to determine inclusion or exclusion, the full texts of the articles were retrieved. In addition to the electronic search, a manual screening of the reference lists and related publications of the selected articles was performed to ensure that no relevant studies were overlooked.

### 2.2. Data Extraction

After selecting the articles to be included in the present narrative review, the following data were extracted and reported in Table 1: first author and year of publication; country; ASD participants; control participants; study design; study aim; social versus non-social nature/content of the task; task/skill taught; and brief results.

## 3. Results

### 3.1. Selected Studies

The literature search generated 40 articles. After the initial screening, 33 studies were retrieved. Finally, 19 studies met our inclusion criteria. Three more studies were identified through the references [18,22,37]; therefore, 22 studies were included in this review (Table 1).

### 3.2. Study Characteristics

#### 3.2.1. Age Characteristics of the Participants of the Included Studies

**Figure 1:**
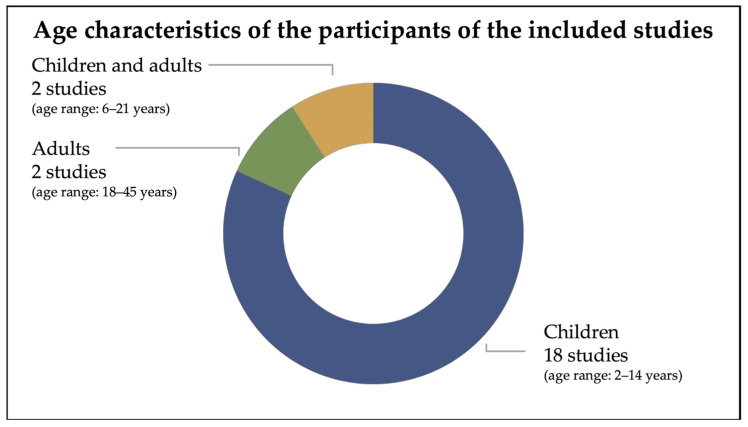
Age characteristics of the participants of the included studies.

The 22 studies included in the present review comprehended both children and/or adults (Figure 1). Specifically, in 18 studies the target age was children (age range: 2–14 years) [18,22,28,30,31,32,33,34,36,37,38,39,40,41,42,43,44,46]; in 2 studies the target age was adults (age range: 18–45 years) [29,47]; in 2 studies the target age included both children and adults (age range: 6–21 years) [35,45]. All details of the studies are reported in Table 1.

#### 3.2.2. Presence/Absence of a Control Group in the Included Studies and Sample Sizes

**Figure 2:**
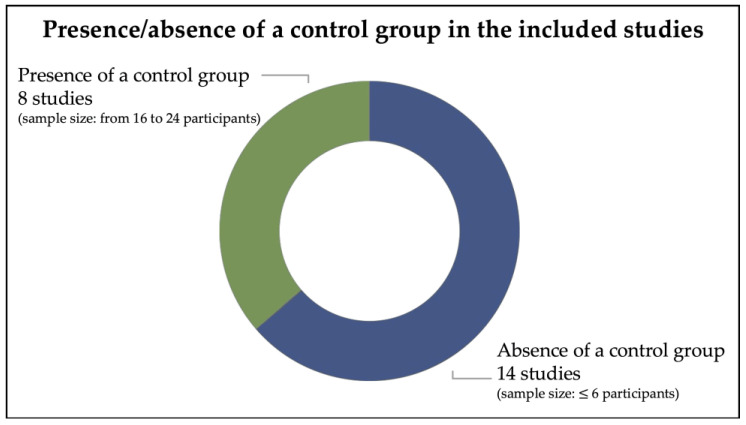
Presence/absence of a control group in the included studies.

Of the 22 studies included in the present review, most lacked a control group (Figure 2). Indeed, 14 studies did not include a control group [18,22,28,30,32,33,34,36,38,39,43,44,45,46], while the remaining 8 included one [29,31,35,37,40,41,42,47]. Specifically, the 14 studies that lacked a control group involved small sample sizes (ranging from 2 to 6 participants with ASD) and employed single-case designs. Conversely, studies that included a control group consistently recruited larger samples (from 16 to 24 participants with ASD) who were matched with an equal or greater number of TD participants. All study details are reported in Table 1.

#### 3.2.3. Cognitive Level of Participants with ASD

**Figure 3:**
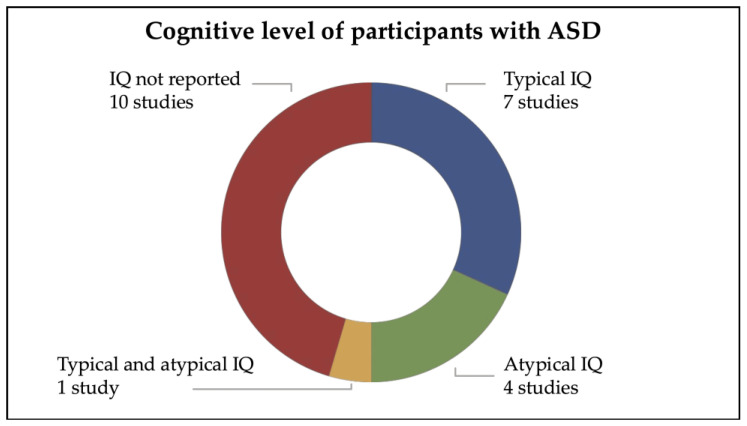
Cognitive level of participants with ASD.

The 22 studies included in the present review comprehended participants with both typical and/or atypical IQ scores (Figure 3). Specifically, 7 studies included participants in the typical IQ range [29,35,37,38,39,40,47], 4 studies included participants in the atypical IQ scores [28,32,41,42], only 1 study included participants with both typical and atypical IQ scores [33], while 10 studies did not report IQ scores [18,22,30,31,34,36,43,44,45,46]. All details of the studies are reported in Table 1.

#### 3.2.4. Skills Taught and/or Components of Observational Learning Assessed

**Figure 4:**
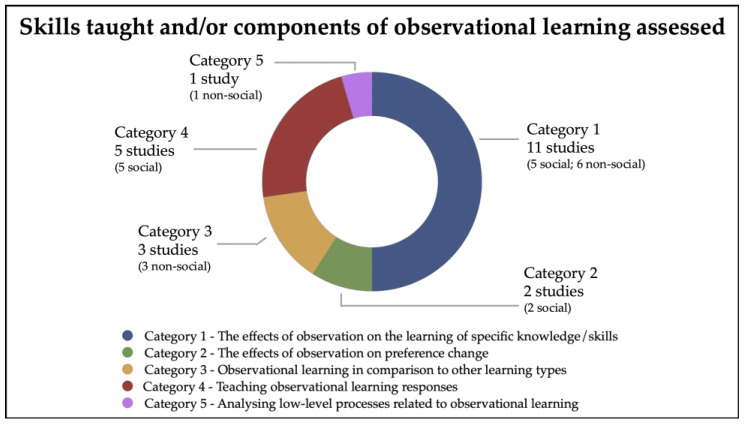
Skills taught and/or components of observational learning assessed.

Considering the 22 included studies as a whole, it clearly emerges that—although all the studies focused on observational learning—they were conducted with specific objectives that differed from one another and could be traced back to different “categories”. Moreover, within each category, studies were classified as social versus non-social, based on the nature/content of the tasks and treatments (Table 1; Figure 4).

Specifically, the following five categories can be identified:

- *Category 1—The effects of observation on the learning of specific knowledge/skills.* Eleven studies aimed to examine the effects of observation in relation to learning specific knowledge/skills. Specifically, five studies were classified as social and addressed the use of observational procedures to teach emotional aspects (the social learning of threat [29]; emotion recognition skills of four basic facial expressions using a robotic social environment [34]; metaphorical statements about emotions [28]) and sight words (through a robotic setting [33] or a standard setting [18]). Conversely, six studies were classified as non-social and addressed the use of observational procedures to teach science facts [30]; motor skills [35,37]; independent access to age-appropriate recreation and leisure skills, that is accessing video games [36]; new actions, that is opening a box in order to take a sweet [31]; stimulus classes, consisting of spoken words and their corresponding pictures and printed words [32].
- *Category 2—The effects of observation on preference change.* Two studies, classified as social, aimed to evaluate the effects of observation on preference change in children with ASD [38,39]; specifically, studies evaluated if the observation was effective in switching preferences from high-preference tangible items to initially low-preference tangible items [38] or to an initially low-preference social activity [39].
- *Category 3—Observational learning in comparison to other learning types.* Three studies, classified as non-social, aimed to compare different types of learning, including observational learning [40,41,42]. Specifically, 2 studies aimed to analyze observational learning and compared it with trial-and-error learning through either a visuo-motor sequence learning task [40] or the building of a house with a set of bricks [41]; while 1 study aimed to compare the following four different learning types: observational learning, instrumental learning, reversal learning, and conditional discriminations [42].
- *Category 4—Teaching observational learning responses*. Five studies, classified as social, aimed to evaluate procedures for teaching observational learning responses, attempting to isolate and train specific components/skills related to observational learning [22,43,44,45,46].
- *Category 5—Analyzing low-level processes related to observational learning.* One study, classified as non-social, aimed to examine low-level processes, namely the contribution of sensorimotor processing during action-observation relating to atypical biological kinematics [47].

## 4. Discussion

The present review aimed to analyze the current state of knowledge on observational learning in individuals with ASD. Specifically, in order to optimize the conceptualization of the results and related insights, the findings from the 22 articles that met the inclusion criteria are discussed below according to the five identified categories.

### 4.1. Category 1—The Effects of Observation on the Learning of Specific Knowledge/Skills

Most of the studies on observational learning included in this review aimed to examine the effects of observation on the acquisition of specific knowledge/skills. In fact, this category includes 11 studies that have used observational procedures to teach very different aspects, which were distinguished in social and non-social. Specifically, the studies classified as social addressed the use of observational procedures to teach emotional aspects [28,29,34] and sight words [18,33]. Conversely, the studies classified as non-social addressed the use of observational procedures to teach science facts [30], motor skills [35,37], independent access to age-appropriate recreation and leisure skills [36], new actions, that is opening a box in order to take a sweet [31], and stimulus classes, consisting of spoken words and their corresponding pictures and printed words [32].

Starting from the results of the five studies regarding the use of social tasks, it emerges that with the exception of one study involving adults and employing a quasi-experimental design [29], the remaining four studies were conducted on children, employed single-case designs with limited sample sizes, and lacked control groups [18,28,33,34]. Regarding the emotional aspects, the three studies were very different in terms of issues addressed and the methodology used [28,29,34]. Specifically, Espinosa and colleagues [29] investigated fear learning through social (observational) and Pavlovian learning paradigms in adults with ASD in the typical IQ range and neurotypical controls. Main results showed that during learning, individuals with ASD attended less to the demonstrator’s face and—when later tested—displayed stronger observational autonomic indices of learning (skin conductance) compared to controls. Conversely, they showed typical fear learning in the Pavlovian paradigm. This specific pattern indicated that enhanced fear learning was specific to social learning and may have resulted from a reduced ability to use facial information to downregulate the emotional response. As suggested by the authors, these findings have important clinical implications. Indeed, individuals with ASD have an increased risk for classical anxiety disorders recognized in the DSM-5, as well as for idiosyncratic fears [48,49]; so, enhanced social fear learning could contribute to the development of these problems, particularly in individuals experiencing difficulties with interpreting others’ reactions and emotions. Still remaining within emotional aspects, a second study—using a multiple-baseline design with a very small sample and lacking a control group—was conducted to evaluate the efficacy of observational learning in teaching children with ASD to state metaphorical statements about emotions when provided a picture, as well as to intraverbally state an appropriate emotion when provided a scenario and corresponding metaphorical emotion [28]. The main results showed that an observational teaching strategy was effective in teaching children with ASD to correctly tact emotions when given metaphors.

Finally, the study of Soleiman and colleagues [34]—that similarly to the previous study employed a multiple-baseline design with a limited sample size and lacked a control group—addressed a very new and intriguing issue, evaluating whether a fully robotic social environment (without human therapist) was effective in teaching children with ASD to recognize and understand the emotions of others through observational learning. More specifically, the authors tried to answer this question: is it possible to teach emotion recognition skills of four basic facial expressions (i.e., happiness, sadness, anger, and fear) that children with ASD have difficulty with [50], using a fully robotic social environment through which the participants observed the social interactions of two robots speaking about these facial expressions? The results showed that children with ASD improved their emotion recognition skills by observing this fully robotic social environment with no direct human intervention. Crucially, this positive effect was maintained even after the intervention period.

Learning environments that include robots have proven effective not only for teaching emotion recognition skills but also sight words [33]. Specifically, Saadatzi and colleagues [33]—adopting a single-case research design with a limited sample size and lacking a control group—implemented an instructional package, combining a virtual teacher and a humanoid robot emulating a peer, to evaluate the explicit acquisition and vicarious learning of sight words instructed to children with ASD and a robot peer. The main results showed that children with ASD could learn the sight words that were instructed to the robot by the virtual teacher through observational learning. Indeed, they not only acquired, maintained, and generalized 100% of the words explicitly instructed to them but, more importantly, they vicariously learned 94% of the words solely instructed to the robot. Another single-case study, using a non-robotic observational learning setting, demonstrated that children with ASD can benefit from observational learning to acquire sight words [18]. Specifically, Ledford and colleagues [18] instructed sight words to six children with ASD in groups of two and demonstrated that five of the participants vicariously learned non-target words taught to their peers. Moreover, more importantly, results during the generalization condition showed that information was maintained over time and generalized to a natural environmental context.

Overall, the findings of the five studies employing social tasks described above are promising. However, they should be interpreted with caution because all studies except one had very small sample sizes, adopted single-case designs, and lacked control groups.

Moving to the results of the six studies regarding the use of non-social tasks, it emerges that half of them employed quasi-experimental or experimental designs, while the other half employed single-case designs with limited sample sizes and lacked control groups. Moreover, with the exception of one study involving both children and adults, the remaining five studies were conducted solely with children. Based on evidence from studies employing quasi-experimental or experimental designs, a beneficial effect of observational learning procedures emerges in teaching children new actions, such as opening a box to obtain a sweet [31], as well as in teaching motor skills to both children and adults [35,37]. These results are in line with those of studies employing single-case designs. Indeed, these latter studies have demonstrated that children with ASD can benefit from observational learning to acquire a scattered set of skills, including science facts [30]; independent access to age-appropriate recreation and leisure skills, such as accessing video games [36]; stimulus classes, consisting of spoken words and their corresponding pictures and printed words [32].

### 4.2. Category 2—The Effects of Observation on Preference Change

The effects of specific observation procedures were also evaluated in relation to the possibility of altering preferences for stimuli or activities in the presence of ASD [38,39]. Before discussing the results in detail, it is important to note that both studies were conducted on children and employed single-case designs with limited sample sizes and lacked control groups; moreover, given the nature/content of the task, both studies were classified as social.

Specifically, these two studies aimed to evaluate whether the observation was effective in shifting preferences from high-preference tangible items to initially low-preference tangible items [38] or to an initially low-preference social activity [39]. The main results showed that observation can be an effective way to broaden the limited and repetitive patterns of behavior, interests, and activities that characterize ASD. Indeed, Leaf and colleagues [38] found that an observational procedure, in which children with ASD observed a preferred adult playing with toys for which the children initially showed low preference, increased their rate of choosing those items. Importantly, this positive effect of the observation was also found for social activities [39]. Indeed, Leaf and colleagues [39] showed that preferences of children with ASD shifted from a highly preferred tangible item to an initially nonpreferred social reinforcer after an observational procedure in which participants simply watched a peer select and engage in social activities.

Although caution is necessary primarily due to the very small sample and the single-case design of both studies, these findings—indicating that the reinforcing values of both tangible items and social activities can be altered—have important implications for therapists, clinicians, and teachers working with this population. Observation procedures may provide an effective way to expand the interests of children with ASD, leading to greater diversity in play, increased engagement in age-appropriate activities, and enhanced social opportunities with peers.

### 4.3. Category 3—Observational Learning in Comparison to Other Learning Types

Another category of studies aimed not only to analyze observational learning but also to compare it with other types of learning. Specifically, of the three studies that fall into this category, two studies aimed to analyze observational learning and compare it with trial-and-error learning, which is the type of learning that occurs through active experience and is typically characterized by a more time-consuming process than observational learning [40,41]. Conversely, the third study aimed to compare four different learning types—that is, observational learning, instrumental learning, reversal learning, and conditional discriminations—often involved in reward-based educational programs [42]. Before discussing the results in detail, it is important to note that all studies were conducted on children, employed quasi-experimental designs, and—given the nature/content of the task—were classified as non-social.

Using a visuo-motor sequence-learning task, Foti and colleagues [40] found that high-functioning children with ASD were as efficient as TD children in the observational learning task, while they were severely impaired in the trial-and-error learning task. More specifically, through an observational training in which the participants observed a model detecting the visuo-motor sequence, children with ASD learned to put into action the correct decision-making strategy and the appropriate strategies to discover rules and generate new knowledge, hence overcoming the executive function deficits that severely impaired their performance in the trial-and-error learning task. However, despite the positive effect of observational training, children with ASD exhibited hyperimitative tendencies, reproducing even the model’s clearly wrong actions. Interestingly, the same propensity to reproduce the model’s correct, incorrect, and irrelevant actions—through an approach defined by the authors as a “copy-all” learning strategy—was also found in a group of low-functioning children with ASD in an observational task that required building a house with a set of bricks [41]. A different pattern of results in terms of observational learning was found in the study by Reed and colleagues [42], in which four different learning types (observational learning, instrumental learning, reversal learning, and conditional discriminations) were compared. Specifically, children with ASD showed impaired performance on the observational learning task. It is important to note that this learning pattern was specific to this population, as indicated by comparison with another clinical population (Down syndrome), which did not present deficits in observational learning, but in instrumental learning.

### 4.4. Category 4—Teaching Observational Learning Responses

Five studies aimed to evaluate procedures for teaching observational learning responses to participants with ASD, attempting to isolate and train specific components/skills related to observational learning [22,43,44,45,46]. Before discussing the results in detail, it is important to note that all studies employed single-case designs with limited sample sizes, lacked control groups, and—given the nature/content of the task—were classified as social. Moreover, with the exception of one study involving both children and adults [45], the remaining four studies were conducted on children [22,43,44,46].

Specifically, MacDonald and Ahearn [45]—following a preliminary assessment that revealed observational learning deficits in a group of participants with ASD—sought to teach observational learning by training specific skills known to be prerequisites for observational learning to occur (i.e., attending, imitation, delayed imitation, consequence discrimination) to produce that performance. The results showed that all participants engaged in observational learning across multiple tasks after training, demonstrating that observational learning can be taught to children with ASD by teaching those prerequisite skills that may be deficient in their repertoire.

In line with these results are those of the study by Taylor and colleagues [46], which aimed to evaluate whether it is possible to facilitate observational learning through a procedure of monitoring responses, consisting of imitating the peer’s response and attending to the instructional materials. Specifically, the effects of this procedure were evaluated on the acquisition of sight words. The main results showed that training monitoring responses positively affected the acquisition of unknown words; indeed, participants read more accurately during the test session when the monitoring responses were required. Importantly, these findings indicate that some responses, if taught to children with ASD, may facilitate learning during observational learning contexts. However, in this study, participants observed only correct responses that were followed by reinforcement; in other words, they were not required to discriminate between reinforced (correct) responses and nonreinforced (incorrect) responses. To address this specific aspect, DeQuinzio and Taylor [43] evaluated the effectiveness of teaching children with ASD to discriminate between the reinforced and nonreinforced responses of an adult model. The results showed that a discrimination training—in which participants were taught, in the presence of target pictures, to imitate the reinforced responses of an adult model and to say “I don’t know” when the model’s responses were not reinforced—permitted the maintenance of the discrimination during the test sessions. This study illustrated the importance of systematic instruction for teaching learners with ASD to make conditional discriminations during observational learning sessions.

Finally, the study by DeQuinzio and colleagues [44]—which is also to be framed within those that evaluate procedures for teaching observational learning responses to children with ASD—expanded Taylor and colleagues [46] and DeQuinzio and Taylor [43] by including both unknown and known stimuli during observational learning sessions. In line with the results of the above-mentioned studies, this study showed that children with ASD can learn complex conditional discriminations through observational learning [44].

Overall, although caution is necessary primarily due to the very small sample sizes and the single-case designs of all studies, the findings from studies falling into this fourth category allowed us to isolate variables and to achieve insights about mechanisms that influence observational learning, providing crucial evidence to implement and refine procedures aimed at improving the observational learning skills of individuals with ASD.

### 4.5. Category 5—Analyzing Low-Level Processes Related to Observational Learning

Only one study addressed the issue of observational learning in ASD by examining low-level processes [47]. Before discussing the results in detail, it is important to note that the study was conducted on adults, employed a quasi-experimental design, and—given the nature/content of the task—was classified as non-social. Specifically, the authors based their work on the premise that the observation and voluntary imitation of biological kinematics displayed by a model underpin the acquisition of new motor skills through sensorimotor processes linking perception with action. Accordingly, differences in voluntary imitation in ASD could be related to sensorimotor processing activity during action observation of biological motion, as well as to how sensorimotor integration processing occurs across imitation attempts. Using an observational practice protocol, Foster and colleagues examined the contribution of sensorimotor processing during action-observation [47]. The results showed that participants with ASD exhibited a similar level of accuracy compared to neurotypical participants when imitating both the temporal duration and the atypical kinematic profile of the observed movement. These findings suggest that lower-level perception-action processes, responsible for encoding biological kinematics during the action-observation phase of imitation, are operational in ASD; in this way, they question the assumption of a global deficit in action-observation network processing that generally affects motor imitation in ASD.

### 4.6. Final General Discussion Considering Findings in All Categories

Considering all five categories above examined, the findings indicated that—despite documented deficits in those that are considered prerequisites for observational learning to occur—observation exerts a strong positive effect on the behavior of individuals with ASD in multifaceted ways across both social and non-social observational learning contexts.

Specifically, studies on the effects of observation on the learning of specific knowledge and skills, including some studies using (quasi)-experimental designs and large samples, seem to support the use of observational learning strategies for individuals with ASD. Regarding the acquisition of emotional aspects, an observational teaching strategy was effective in teaching children with ASD to correctly tact emotions when given metaphors [28]. Coherently, Soleiman and colleagues [34] proved that a human-free robotic social environment led to durable improvements in emotion recognition skills of four basic facial expressions (i.e., happiness, sadness, anger, and fear) that children with autism have difficulty with. On the other hand, in the study by Espinosa and colleagues [29], less adaptive effects of observation were found in relation to fear learning. Indeed, individuals with ASD exhibited stronger fear learning exclusively in the social (observational) learning paradigm, but not in the Pavlovian one, coupled with less attention to the demonstrator’s face [29]. Continuing to consider this first category of studies, it emerges that observation could be an effective learning strategy for facilitating—and often maintaining and generalizing—the acquisition of academic, motor, and recreational skills. In terms of academic skills, children with ASD may benefit from observational learning settings to acquire, maintain, and generalize sight words taught by a robot [33] or by their peers [18]. Similarly, positive effects were found in relation to the acquisition of science facts [30] and stimulus classes consisting of spoken words and their corresponding pictures and printed words [32]. Furthermore, the effects of observation have also been promising in terms of the acquisition of general motor skills [35,37] and new specific actions, such as opening a box to get a sweet [31]. These latest results align well with the findings of Foster and colleagues [47] regarding low-level sensorimotor processes. Indeed, the results showed that participants with ASD exhibited a similar level of accuracy compared to neurotypical participants in imitating both the temporal duration and atypical kinematic profile of the observed movement, suggesting that lower-level perception-action processes, responsible for encoding biological kinematics during the action-observation phase of imitation, were operational in ASD [47].

Other crucial insights come from studies in the second category, which aimed to evaluate whether the observation was effective in switching preferences for stimuli or activities in children with ASD [38,39]. Although the results of both studies should be interpreted with caution because they employed single-case designs with limited sample sizes, they showed that observation can be an effective way to broaden the limited and repetitive patterns of behavior, interests, and activities that characterize ASD, leading to greater diversity in play, increased engagement in age-appropriate activities, and enhanced social opportunities with peers.

Considering the third category of studies comparing observational learning to other learning types, significant evidence emerges regarding the specific features of observational learning [40,41,42]. Indeed, although results on the integrity of observational learning are mixed, the studies—all using (quasi-)experimental designs and large samples—converge in finding a propensity to hyperimitate which leads individuals with ASD to reproduce even the model’s clearly wrong actions. However, this specific characteristic was positively correlated with goal achievement, thus resulting in a functional “copy-all” learning strategy.

Finally, crucial new insights also come from studies of the fourth category that attempt to isolate and train specific components and skills for teaching observational learning responses to individuals with ASD [22,43,44,45,46]. Although all studies involved single-case designs and very small samples, the main findings agree in showing that observational learning can be facilitated and taught to individuals with ASD by teaching those prerequisite skills that may be deficient in their repertoires (i.e., attending, imitation, delayed imitation, consequence discrimination). By doing so, these findings provide new insights into the mechanisms that influence observational learning and, consequently, provide crucial evidence for implementing and refining procedures aimed at improving the observational learning skills of individuals with ASD.

### 4.7. Applicative Implications of the Present Findings

These findings support the use of observational learning strategies for individuals with ASD. However, from an applicative perspective, how can these results inform clinical practice, educational settings, intervention development, and future research?

A primary significant implication concerns the restricted, repetitive, and stereotyped behaviors that characterize individuals with ASD, which can hinder the identification of effective reinforcers, thereby limiting educational and social opportunities. Crucially, the present results suggest that observation can indeed be an effective procedure for altering the reinforcing values of both tangible items and social activities, thus enhancing preferences for previously neutral or non-preferred stimuli and expanding interests in the presence of ASD. This may also have a positive impact on clinical practice, because the restricted and repetitive interests of individuals with ASD can make it difficult for clinicians to find a variety of reinforcers to use when implementing interventions.

A further important implication is related to what can be learned using observational learning procedures. The findings suggested that observation is an effective learning strategy for facilitating—and often maintaining and generalizing—the acquisition of emotional, academic, motor, and recreational skills.

Moreover, considering the crucial role played by observational learning in developing complex abilities—such as language and social responsiveness, with which individuals with ASD often have difficulties—a further implication concerns how to facilitate observational learning. Regarding this specific aspect, the present findings suggest that the engagement of individuals with ASD in observational learning can be facilitated by specific training that teaches the necessary prerequisites for observational learning to occur. In this way, the present findings provide crucial elements to refine procedures aimed at improving engagement in observational learning in the presence of atypical social functioning.

Overall, these findings facilitate the identification of strategies for shifting from one-to-one instruction to group-based social settings across various contexts.

### 4.8. Limitations and Future Research Directions

Despite the important implications of this narrative review for educational, clinical, and social interventions targeting individuals with ASD, several limitations should be acknowledged. Indeed, although this review summarizes the most relevant studies on observational learning in ASD, a limitation concerns the narrative approach and the use of a single database. Consequently, this review should be primarily intended as a theoretical and conceptual overview of observational learning in ASD, rather than as a comprehensive systematic synthesis. A second limitation concerns the representativeness of the available evidence. Indeed, most of the included studies employed single-case designs, recruited very small samples, and reported descriptive indices without inferential statistics. Furthermore, the studies were often conducted with different objectives and used widely varying tools to assess observational learning. All this did not facilitate comparisons among evidence and quantitative synthesis, requiring caution in interpreting the findings until they are replicated in studies adopting experimental designs with larger samples. To overcome these limitations, future research should adopt standardized assessment protocols and transparent statistical reporting practices to facilitate quantitative synthesis and improve comparability across studies. Finally, most of the included studies were mainly conducted on children, thereby restricting the possibility of identifying developmental lifespan trajectories and limiting the generalizability of the results across ages. A larger number of studies involving adults, as well as longitudinal research, are therefore needed to understand the development of observational learning over time, identify optimal intervention periods, and clarify long-term implications for learning outcomes.

## 5. Conclusions

The present narrative review investigated literature pertaining to the complex and timely issue of observational learning in ASD, contributing to achieving a better understanding of the mechanisms underlying social and non-social observational learning and offering new insights into the interplay between observational learning and social information processing. Although caution is warranted due to the heterogeneity in how studies were implemented and their effects assessed, the overall results highlight promising prospects with significant educational, clinical, social, and economic implications, supporting the use of observational learning strategies in both social and non-social skills. Indeed, the core findings indicate that individuals with ASD may be able to learn by observing others, especially if they are taught the prerequisites for observational learning, which may be lacking in their repertoire. Importantly, this could lead to greater social and educational opportunities and reduce reliance on expensive one-on-one instruction. Furthermore, the findings also indicate that observation may provide an effective way to expand the typically restricted and circumscribed interests of children with ASD as well as to increase emotion recognition skills, providing evidence of the significance of observational procedures in improving some of the core deficits of ASD. Future research is warranted to further evaluate variables that may influence observational learning and pioneering research on the use of virtual teaching and robotic social environments.

## Figures and Tables

**Table 1:** Main characteristics and brief results of the studies included in this review.

**Category 1—The Effects of Observation on the Learning of Specific Knowledge/Skills**								
**First Author** **and Year**	**Country**	**ASD** **Participants**	**Control** **Participants**	**Study Design**	**Study Aim**	**Social vs.** **Non-Social** **Nature/Content of the Task**	**Task/Skill Taught**	**Brief Results**
Dixon et al., 2017 [28]	USA	3 children with ASD 3 m Age range: 10–11 years Participant 1: 11-year-old IQ: 70 Participant 2: 10-year-old IQ: 71 Participant 3: 11-year-old IQ: 47	-	Multiple-baseline design across participants	To evaluate the efficacy of observational learning using the rival-model technique in teaching children with ASD to state metaphorical statements about emotions when provided a picture, as well as to intraverbally state an appropriate emotion when provided a scenario and corresponding metaphorical emotion.	Social	Teaching to state metaphorical statements about emotions.	Observational teaching strategy was effective in teaching children with ASD to correctly tact emotions when given metaphors.
Espinosa et al., 2020 [29]	Sweden	23 adults with ASD 14 f; 9 m Age: 31.61 ± 9.50 IQ: 108.7 ± 20.13	25 neurotypical controls 9 f; 16 m Age: 26 ± 4.90 IQ: 111.2 ± 19	Quasi-experimental within-groups design.	To examine threat learning through social (observational) and Pavlovian learning paradigms in individuals with ASD and neurotypical controls.	Social	Social learning of threat.	In comparison to controls, individuals with ASD displayed stronger learned defensive responses (skin conductance) when learning was conveyed solely through observation. Moreover, eye-tracking analyses showed that individuals with ASD attended less to the demonstrator’s face than controls. Conversely, individuals with ASD showed typical fear learning in the Pavlovian conditioning task, suggesting that enhanced fear learning was relatively specific for social learning.
Kiyak & Toper, 2022 [30]	Turkey	3 participants with ASD 3 m Age range: 13–14 years	-	Multiple-probe design across participant dyads.	To examine the effects of the simultaneous prompting procedure and observational learning in teaching science facts to middle-school students with ASD, using Zoom.	Non-social (specifically, academic skills)	Science facts	Simultaneous prompting procedure delivered via telehealth was effective in teaching science facts to middle-school students with ASD. In addition to this, when the participants were not directly exposed to the intervention, but observed teaching sessions, they still acquired the targets at a similar rate as the participants exposed to training.
Ledford et al., 2008 [18]	USA	6 children with ASD 6 m Age range: 5.9–8.4 years Participant 1: 5 y 9 m old CARS: 41 PER-R: 34 months Participant 2: 5 y 9 m old CARS: 41 PER-R: 32 months Participant 3: 6 y 3 m old CARS: 43.5 PER-R: 36 months Participant 4: 8 y 3 m old CARS: 36 PER-R: 46 months Participant 5: 6 y 9 m old CARS: 35 PER-R: 41 months Participant 6: 8 y 4 m old CARS: 36 PER-R: 38 months	-	A multiple-probe design across behaviors.	To evaluate the acquisition of incidental and observational information presented to 6 children with ASD in a small group instructional arrangement, using a constant time delay (CTD) procedure.	Social	Sight words	Children with ASD learn high percentages of observational and incidental information. Moreover, results during the generalization condition show information was maintained across time and generalized to a natural environmental context.
Nadel et al., 2011 [31]	France	20 nonverbal children with ASD with a developmental age between 24 and 36 months. Chronological age range: 35–104 months	20 TD children matched with the ASD group based on developmental age. Chronological age range: 22–40 months	Quasi-experimental between-groups design.	To analyze whether low-functioning children with ASD can learn novel actions via observation.	Non-social	Learning new actions: specifically, participants had to open an experimental box that required them to perform a hierarchical sequence of subgoals in order to take a sweet from the box.	Only the TD children learned the sequence of subgoals after the first video-demonstration. Conversely, children with ASD needed a second demonstration to learn the sequence of subgoals.
Rehfeldt et al., 2003 [32]	USA	Experiment 2: 3 children with ASD 3 m Age range: 7–9 years Participant 1: 9-year-old IQ: 77 Participant 2: 9-year-old IQ: 70 Participant 3: 7-year-old IQ: 70 Experiment 1 was omitted because it did not include participants with ASD.	-	A pre-test post-test design	To determine whether children with ASD would demonstrate stimulus classes consisting of spoken words and their corresponding pictures and printed words, after observing a model demonstrate the prerequisite conditional discriminations.	Non-social	The formation of stimulus classes.	Participants may acquire stimulus classes consisting of spoken words, pictures, and printed words after observing a model demonstrate accurate conditional discriminations among the stimuli.
Saadatzi et al., 2018 [33]	USA	3 children with ASD 3 m Age range: 6–8 years Participant 1: 8-year-old IQ: 82 CARS: 38 Participant 2: 8-year-old Verbal IQ: 75 Performance IQ: 49 CARS: 42 Participant 3: 6-year-old IQ: 107	-	Multiple-probe design across participants.	To evaluate the effects of the instructional package (combining a virtual teacher and a humanoid robot emulating a peer, resembling a small-group arrangement) on the explicit acquisition and vicarious learning of sight words instructed to 3 children with ASD and a robot peer.	Social in a virtual and robotic instructional package	Sight words	Participants acquired, maintained, and generalized 100% of the words explicitly instructed to them, made fewer errors while learning the words common between them and the robot peer, and vicariously learned 94% of the words solely instructed to the robot.
Soleiman et al., 2023 [34]	Iran	5 children with ASD (4 with a diagnosis of severity level 1, requiring minimal support; 1 with a diagnosis of severity level 2, requiring substantial support) and 1 with a developmental delay diagnosis. 1 f; 5 m Age range: 6–11 years	-	A single case A-B-A design experiment with a generalization probe across novel stimuli and settings.	To evaluate whether a fully robotic social environment designed to simulate the social environment for children (without a human therapist) is effective in teaching children with ASD to recognize and understand the emotions of others through observational learning.	Social in a fully robotic environment	Emotion recognition skills of four basic facial expressions (i.e., happiness, sadness, anger, and fear) by observing the social interactions of two robots speaking about these facial expressions.	Children improved their emotion recognition skills and, importantly, were able to generalize and maintain them after the intervention period.
Sotoodeh & Taheri-Torbati, 2021 [35]	Iran	24 participants with ASD. 3 f; 21 m Age range: 6–17 years IQ: 97.12 ± 7.47	24 age-matched TD participants. 3 f; 21 m Age range: - IQ: 102 ± 6.17	Randomized Controlled Trial	To compare point-light display (PLD) to video observation as instructional models for teaching motor skills to children with ASD versus TD participants.	Non-social	Motor skills	Participants with ASD learning through PLD performed better than participants with ASD learning through video modeling (VM) in the acquisition phase and at retention and transfer testing. Conversely, participants in the TD group did not show any learning differences between PLD and VM training conditions. Finally, gaze recordings revealed that participants with ASD paid more attention to relevant demonstration points in the PLD than in the VM condition.
Spriggs et al., 2016 [36]	USA	4 children with ASD 1 f; 3 m Age range: 8–11 years Participant 1: 11-year-old male WPPSI-III: “un-testable” CARS: 39.5 GARS-2: 126 Participant 2: 11-year-old male GARS-2: 85 (parent), 89 (teacher) SCQ: 33 Participant 3: 10-year-old male GARS-2: 117 Participant 4: 8-year-old female GARS-2: 122	-	Multiple probe design across participants	To evaluate both video modeling and observational learning to teach age-appropriate recreation and leisure skills (i.e., accessing video games) to children with ASD.	Non-social	Independent access to age-appropriate recreation and leisure skills (i.e., accessing video games).	A functional relation was established between video modeling and independent access to various gaming devices/video games for three students; observational learning was effective to increase independently completed steps.
Taheri-Torbati & Sotoodeh, 2018 [37]	Iran	24 children with ASD 24 m Age range: 9–13 years CA: 10.8 ± 1.5 Nonverbal IQ: 93.6 ± 8.8 GARS: 94.2 ± 11.9	24 TD children matched with the ASD group based on age, height, and weight. CA: 10.9 ± 1.3 Nonverbal IQ: 97.5 ± 5.5	Quasi-experimental between-groups design.	To investigate the effect of two kinds of demonstration, namely video and live modeling on learning of motor skills in children with ASD.	Non-social	Motor skills	Both video and live modeling methods positively impacted the acquisition of motor skills in children with ASD. These results indicated that modeling is an effective technique for teaching and improving motor skills learning in this population.
**Category 2—The Effects of Observation on Preference Change**								
**First Author** **and Year**	**Country**	**ASD** **Participants**	**Control** **Participants**	**Study Design**	**Study Aim**	**Social vs.** **Non-Social** **Nature/Content of the Task**	**Task/Skill Taught**	**Main Results**
Leaf et al., 2012 [38]	USA	3 children with ASD 3 m Age range: 5–6 years Participant 1: 5-year-old IQ: 117 SSRS-P: 67 Participant 2: 6-year-old IQ: 87 SSRS-P: 63 Participant 3: 5-year-old IQ: 89 SSRS-P: 106	-	An ABABA design or an ABACA design was used to evaluate the effects of the intervention.	To evaluate the observational effects on the preferences of children with ASD. More specifically, to examine whether observation of a known and preferred adult playing with low-preference toys in presumably novel and exciting ways could increase the rate of choosing those items by children with ASD.	Social	To shift the children’s preferences from a highly preferred item to a less preferred one.	The observation procedure was effective in switching preferences from high-preference tangible items to initially low-preference tangible items for children with ASD.
Leaf et al., 2016 [39]	USA	3 children with ASD 1 f; 2 m Age: 5 years Participant 1: 5-year-old IQ: 129 VABS: 78 SSRS-P: 90 Participant 2: 5-year-old IQ: 85 VABS: 73 SSRS-P: 92 Participant 3: 5-year-old IQ: 102 SSRS-P: 81	-	Reversal design: ABAB, ABACAB, or ABACABCBCBCB design.	To shift children’s preferences from a highly preferred tangible item to an initially nonpreferred social reinforcer using an observational conditioning procedure.	Social	Preferences for social activities.	The preferences of all three participants shifted from a high-preference tangible item to an initially low-preference social activity simply by watching a peer select and engage in the social activity. These findings provide support for the use of observational procedures to alter preferences.
**Category 3—Observational Learning in Comparison to Other Learning Types**								
**First author** **and Year**	**Country**	**ASD** **Participants**	**Control** **Participants**	**Study Design**	**Study Aim**	**Social vs.** **Non-Social** **Nature/Content of the Task**	**Task/Skill Taught**	**Main Results**
Foti et al., 2014 [40]	Italy	20 children with ASD 2 f; 18 m CA: 10.5 ± 0.07 IQ: 105.85 ± 2.77 ADOS total score: 13.5 ± 0.85	20 TD children matched with the ASD group based on CA, IQ, and gender. 2 f; 18 m CA: 10.05 ± 0.06 IQ: 108 ± 2.36	Quasi-experimental between-groups design	To clarify the features of observational learning in children with ASD and compare them with trial-and-error learning.	Non-social	A task of learning a visuomotor sequence.	Children with ASD were as efficient as TD children in observational learning, while they were impaired in trial-and-error learning. In spite of the beneficial effects of the observational training, children with ASD also reproduced the model’s wrong actions, thus showing marked hyper-imitative tendencies.
Foti et al., 2019 [41]	Italy	16 low-functioning children with ASD. 2 f; 14 m CA: 88.2 m. ± 33.3 MA: 48.4 m. ± 15.4 IQ: 67 ± 21.7	16 TD children matched with ASD group based on MA and gender. 2 f; 14 m CA: 49.3 m. ± 13.9 MA: 47.2 m. ± 14.7 IQ: 103.4 ± 7.9	Quasi-experimental between-groups design	To analyze observational learning in children with ASD and compare it with trial-and-error learning, by means of a behavioral and neuroimaging approach.	Non-social	To build a house with a set of bricks after a video-demonstration and, subsequently, a different house by trial and error.	Children with ASD exhibited deficits in both observational and trial-and-error learning tasks, although their performance improved over time. Moreover, they demonstrated hyper-imitative tendencies that facilitated goal achievement and were associated with areas belonging to mirror neuron system.
Reed et al., 2011 [42]	United Kingdom	20 children with ASD 3 f; 17 m 20 with DS 5 f; 15 m ASD group -mean age of 12.2 years (±1.3, range 11–16); -mean non-verbal mental age equivalent of 9.4 years (±0.8, range 8–11) as measured by Leiter-R; -mean verbal mental age of 9.7 years (±1.0, range 7.5–11.8), giving an approximate mean verbal IQ of 77.4 (±9.1, range 59.4–90.9), as measured by BPVS. DS group -mean age of 13.1 years (±1.6, range 10–16); -mean non-verbal mental age equivalent of 9.6 years (±0.7, range 8–11) as measured by Leiter-R; -mean verbal mental age of 9.6 years (±0.9, range 8.3–11.8), giving an approximate mean verbal IQ of 74.1 (±9.5, range 60.7–97.5), as measured by the BPVS.	20 TD children matched with both clinical groups based on MA. 6 f; 14 m -mean age of 9.6 years (±1.2, range 8–12); -mean non-verbal mental age equivalent of 9.7 years (±1.2, range 8–12); -mean verbal mental age of 9.3 years (±0.9, range 8–11.5), giving an approximate mean verbal IQ of 97.4 (±9.9, range 82.2–128.1), as measured by the BPVS.	Quasi-experimental between-groups design	To investigate the relative abilities of individuals with ASD and DS across four different learning tasks: observational learning, instrumental learning, reversal learning, and conditional discriminations.	Non-social	Four different learning tasks: observational learning, instrumental learning, reversal learning, and conditional discriminations.	ASD group performed worse than the other two groups on the observational learning and conditional discrimination tasks, while the DS group performed worse than the other two groups on the instrumental learning task.
**Category 4—Teaching Observational Learning Responses**								
**First Author** **and Year**	**Country**	**ASD** **Participants**	**Control** **Participants**	**Study Design**	**Study Aim**	**Social vs.** **Non-Social** **Nature/Content of the Task**	**Task/Skill Taught**	**Main Results**
Delgado & Greer, 2009 [22]	USA	2 children with ASD 2 m Age: 5-year-old	-	A delayed multiple-probe design across participants.	To evaluate the effects of teaching peer monitoring on the emergence of an observational learning capability.	Social	Experiment 1: textual responses and pictures. Experiment 2: vocal spelling responses.	Observational learning emerged in all participants following the peer monitoring intervention. Consequently, participants showed a higher level of correct responses when observing their peers.
DeQuinzio & Taylor, 2015 [43]	USA	4 children with ASD 3 f; 1 m Age range: 6–12 years Participant 1: 6-year-old Age-equivalent score on the PPVT: 4 y. 10 m. Age-equivalent score of the EVT: 5 y. 3 m. Participant 2: 9-year-old Age-equivalent score on the PPVT: 4 y. 9 m. Age-equivalent score of the EVT: 4 y. 4 m. Participant 3: 12-year-old Age-equivalent score on the PPVT: 5 y. 5 m. Age-equivalent score of the EVT: 5 y. 4 m. Participant 4: 7-year-old Age-equivalent score on the PPVT: 6 y. 3 m. Age-equivalent score of the EVT: 6 y. 4 m.	-	A multiple baseline design across participants.	To evaluate the effectiveness of teaching children with ASD to discriminate (through a discrimination training) between the reinforced and non-reinforced responses of an adult model.	Social	To discriminate the reinforced and nonreinforced responses of an adult model.	The discrimination training—in which participants were taught, in the presence of target pictures, to imitate the reinforced responses of an adult model and to say “I don’t know” when the model’s responses were not reinforced—permitted the maintenance of the discrimination during the test sessions.
DeQuinzio et al., 2018 [44]	USA	3 children with ASD 3 m Age range: 7–12 years Participant 1: 7-year-old Age-equivalent score on the PPVT-4: 6 y. 3 m. Age equivalent score on the EVT-II: 5 y. 11 m. Participant 2: 12-year-old Age-equivalent score on the PPVT-4: 7 y. 10 m. Age equivalent score on the EVT-II: 5 y. 8 m. Participant 3: 12-year-old Age-equivalent score on the PPVT-4: 4 y. 3 m. Age equivalent score on the EVT-II: 6 y.	-	Multiple-baseline design across participants	To evaluate the effects of discrimination training on the discrimination of consequences applied to modeled responses using both known and unknown pictures during observational learning sessions.	Social	To label pictures	Test sessions measured responding to known and unknown pictures and showed acquisition over baseline levels.
MacDonald & Ahearn, 2015 [45]	USA	6 participants with ASD 2 f; 4 m Age range: 8–21 years Participant 1: 16-year-old boy PPVT-4B: - Participant 2: 8-year-old boy PPVT-4B: 2 y. 7 m. Participant 3: 14-year-old girl PPVT-4B: - Participant 4: 10-year-old boy PPVT-4B: - Participant 5: 21-year-old woman PPVT-4B: 6 y. 8 m. Participant 6: 19-year-old man PPVT-4B: 5 y. 7 m.	-	Multiple-probe design across and/or within participants.	To develop an assessment to test for the presence of observational learning (OL) across a variety of tasks, including academic and leisure activities. If OL was deficient, authors sought to teach it by training specific skills (i.e., attending, imitation, delayed imitation, consequence discrimination) to produce that performance. Then, generalization of OL was assessed following training on different tasks, variations on that type of task, or both.	Social	Academic and leisure activities; specifically, hidden items, computer games, academic, construction toys, and building toys.	All six participants exhibited deficits in OL. After training, 5 of the 6 participants engaged in OL across multiple tasks and task variations, demonstrating generalization. For 1 participant, generalization of performance did not occur across tasks but did occur within task variations. This study demonstrated that OL can be taught to children with ASD across a variety of leisure and academic tasks.
Taylor et al., 2012 [46]	USA	3 children with ASD 1 f; 2 m Age range: 3.8–4.8 years Participant 1: 4.5 years PPVT: <1.9 years Participant 2: 4.8 years PPVT: 2 years Participant 3: 3.8 years PPVT: <1.9 years	-	Within-subjects design	To evaluate the effects of monitoring peers’ reading responses on the acquisition of sight words during an observational learning procedure.	Social	Sight words	Monitoring responses positively affected the acquisition of unknown words.
**Category 5—Analyzing Low-Level Processes Related to Observational Learning**								
**First Author** **and Year**	**Country**	**ASD** **Participants**	**Control** **Participants**	**Study Design**	**Study Aim**	**Social vs.** **Non-Social Nature/Content of the Task**	**Task/Skill Taught**	**Main Results**
Foster et al., 2023 [47]	UK	20 adult participants with ASD without language or cognitive impairment. 2 f; 18 m CA: 25 ± 7; range: 18–44 Full scale IQ: 107 ± 9; range: 91–125 Verbal IQ: 106 ± 11; range: 88–130 Performance IQ: 106 ± 11; range: 82–128	20 neurotypical adult participants matched with the ASD group based on CA, IQ, and gender. 2 f; 18 m CA: 25 ± 7; range: 18–45 Full scale IQ: 109 ± 8; range: 94–123 Verbal IQ: 109 ± 8; range: 96–125 Performance IQ: 107 ± 12; range: 82–128	Quasi-experimental between-groups design	To investigate the operational nature of processes underpinning the representation of atypical biological kinematics in ASD, using an observational practice protocol.	Non-social	During the test session, participants recalled and reproduced the movement displayed by the atypical model during observational practice.	Participants with ASD imitated both the temporal duration and atypical kinematic profile of the observed movement with a similar level of accuracy as neurotypical participants. These findings suggest the lower-level perception-action processes responsible for encoding biological kinematics during the action observation phase of imitation are operational in ASD.

ASD: autism spectrum disorder; BPVS: British Picture Vocabulary Scale; CA: chronological age; CARS: Childhood Autism Rating Scale; DS: Down syndrome; EVT-II: Expressive Vocabulary Test; GARS-2: Gilliam Autism Rating Scale, Second Edition; MA: mental age; PEP-R: Psychoeducational Profile (Revised); PPVT: Peabody Picture Vocabulary Test; SCQ: Social Communication Questionnaire; SSRS-P: Social Skills Rating Scale Parent; TD: typically developing; VABS: Vineland Adaptive Behavior Composite Score; WPPSI-III: Wechsler Preschool and Primary Scale of Intelligence, Third Edition.

## Data Availability

No new data were created or analyzed in this study. Data sharing does not apply to this article.

## Abbreviations

The following abbreviations are used in this manuscript:

ASD	Autism Spectrum Disorder
TD	Typically Developed
